# Safety Profile and Hepatotoxicity of Anaplastic Lymphoma Kinase Tyrosine Kinase Inhibitors: A Disproportionality Analysis Based on FDA Adverse Event Reporting System Database

**DOI:** 10.3390/toxics13030210

**Published:** 2025-03-14

**Authors:** Yun Yang, Shiyi Tan, Yuepu Pu, Juan Zhang

**Affiliations:** 1 Key Laboratory of Environmental Medicine Engineering, Ministry of Education of China, School of Public Health, Southeast University, Nanjing 210009, China; yunyang1108@163.com (Y.Y.); tanshiyicc@163.com (S.T.); yppu@seu.edu.cn (Y.P.); 2 Jiangsu Institute for Sports and Health (JISH), Nanjing 211100, China

**Keywords:** ALK-TKIs, pharmacovigilance, adverse events, hepatotoxicity, U.S. FDA adverse event reporting system

## Abstract

Anaplastic lymphoma kinase tyrosine kinase inhibitors (ALK-TKIs) have become first-line therapies for advanced non-small cell lung cancer (NSCLC) with ALK rearrangements. This study investigates ALK-TKI-associated adverse events (AEs), focusing on identifying hepatotoxicity signals and previously undocumented safety concerns. Using disproportionality analysis of 56,864 reports from the FDA Adverse Event Reporting System (FAERS) database, we systematically classified AEs via the Medical Dictionary for Regulatory Activities (MedDRA). At the System Organ Class (SOC) level, crizotinib exhibited a significantly stronger signal for eye disorders, ceritinib was uniquely linked to gastrointestinal disorders, and loratinib was predominantly associated with metabolism and nutrition disorders. Several AEs previously undocumented in drug labels were identified, including pericardial effusion, elevated C-reactive protein, hemolytic anemia, hemoptysis, and decreased hemoglobin. Furthermore, crizotinib, ceritinib, and alectinib were significantly associated with hepatotoxicity, marked by elevated alanine aminotransferase, aspartate aminotransferase, and hepatic enzyme levels. These findings highlight the need for vigilant monitoring of unlabeled AEs and potential label updates, particularly for hepatotoxicity risks associated with crizotinib, ceritinib, and alectinib.

## 1. Introduction

Lung cancer remains a global health challenge, ranking among the most frequently diagnosed malignancies and representing the leading cause of cancer-related mortality worldwide. According to epidemiological data, approximately 2 million new cases and 1.76 million deaths are reported annually [1], with non-small cell lung cancer (NSCLC) being the predominant histological subtype accounting for 85% of all diagnoses [2]. While platinum-based chemotherapy has been the mainstay treatment [3], the development of molecularly targeted therapies, particularly tyrosine kinase inhibitors (TKIs), has revolutionized treatment paradigms and significantly improved patient outcomes following the discovery of actionable driver mutations [4].

Anaplastic lymphoma kinase (ALK) rearrangements were initially identified in NSCLC patients in 2007, paving the way for ALK-TKI therapies [5]. Approximately 3–7% (more than 60,000 cases annually) of NSCLC patients involve ALK gene rearrangements, more frequently found in adenocarcinoma cases and non-smokers or light smokers [5,6,7]. Since crizotinib’s approval in 2011, successive generations of ALK-TKIs have been developed and were found to transform clinical outcomes: second-generation ceritinib (2014), alectinib (2015), brigatinib (2017), and third-generation lorlatinib (2018) [8]. The National Comprehensive Cancer Network (NCCN) now recommends ALK-TKIs as a first-line therapy for ALK-positive advanced NSCLC [9]. Nevertheless, their clinical utility is tempered by safety concerns, with prevalent adverse events (AEs) spanning gastrointestinal disturbances, hepatotoxicity, visual abnormalities, and pulmonary complications [10]. In the context of the limited accessibility to comprehensive safety data, it is of the utmost importance to conduct a thorough analysis of the overall safety profile associated with ALK-TKIs.

Notably, drug-induced liver injury (DILI), characterized by asymptomatic transaminase elevation or overt hepatic dysfunction, constitutes a critical safety issue, accounting for >50% of acute liver failure cases in Western populations [11]. While hepatotoxicity has been reported in clinical trials [12,13,14,15,16], a comprehensive real-world comparison of ALK-TKI-associated hepatic safety profiles remains conspicuously absent. To our understanding, no prior real-world study has exhaustively delineated and compared the AEs linked to hepatotoxicity induced by ALK-TKIs.

The FDA Adverse Event Reporting System (FAERS), a post-marketing surveillance database aggregating spontaneous AE reports, provides an invaluable resource for pharmacovigilance research. To address existing knowledge gaps, this study conducts a disproportionality analysis of FAERS data to (1) systematically characterize hepatotoxicity signals associated with ALK-TKIs, and (2) identify previously unlabeled AEs requiring clinical attention. Our findings aim to optimize risk-benefit assessments and improve evidence-based monitoring strategies for ALK-TKI therapy.

## 2. Materials and Methods

### 2.1. Data Source

The FAERS database, a publicly accessible repository of post-marketing safety reports, was selected for this retrospective pharmacovigilance study owing to its comprehensive real-world data on adverse drug events. Data spanning from the first quarter of 2011 to the fourth quarter of 2023 were retrieved in the American Standard Code for Information Interchange (ASCII) format. The drug entities were identified using both generic and brand names, with the inclusion criteria restricted to agents approved by the FDA within the study timeframe to ensure temporal relevance.

### 2.2. Data Processing

**Figure 1:**
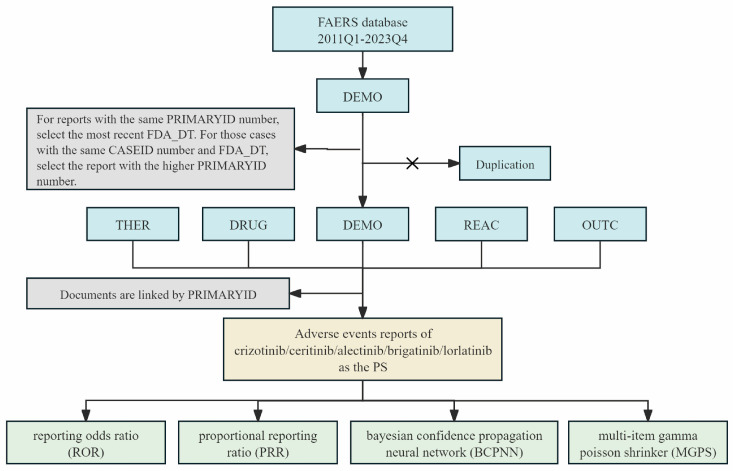
The flow diagram showing the analysis process of the study. “×” indicates the removal of duplicate reports.

The datasets employed in this study encompassed Drug Information (DRUG), Demographic Information (DEMO), Patient Outcomes (OUTC), Indications (INDI), Adverse Reactions (REAC), and Therapy Start and End Dates (THER). The datasets were merged using the PRIMARYID key (Figure 1) and deduplicated following FDA recommendations. (1) We selected the latest FDA_DT as the time identifier when the PRIMARYID was the same. (2) To eliminate duplicate reports submitted by various individuals and institutions, we prioritized the higher PRIMARYID when both FDA_DT and CASEID were the same. The AEs were systematically coded using Medical Dictionary for Regulatory Activities (MedDRA) Preferred Terms (PTs) and mapped to the corresponding System Organ Classes (SOCs) [17]. The clinical characteristics of patients experiencing AEs, including age, gender, reporter type, geographic origin, and patient outcomes were extracted to provide a more comprehensive understanding of the safety profile of the five drugs.

For hepatotoxicity analysis, prior to initiating the drug search, the REAC dataset was standardized by consolidating all liver injury-related preferred terms to the unified designation “DILI.” Table S1 presents the lexicon of liver injury preferred terms derived from the Medical Dictionary for Regulatory Activities (MedDRA), adapted from previous studies investigating antifungal agents or elexacaftor/tezacaftor/ivacaftor and DILI [18,19].

### 2.3. Statistical Analysis

In pharmacovigilance research, disproportionality analysis serves as a key tool to assess potential associations between specific AEs and particular drugs [20]. A 2 × 2 contingency table (Table S2) compared the observed AE frequencies in ALK-TKIs recipients against the expected background rates in the full FAERS dataset. The disproportionality analysis method for AE signal detection includes the Proportional Reporting Ratio (PRR), Reporting Odds Ratio (ROR), Bayesian Confidence Propagation Neural Network (BCPNN), and Multi-item Gamma Poisson Shrinker (MGPS) method. The thresholds for signal detection followed the established criteria (Table S3). The ROR and PRR methodologies are highly efficient in identifying the relative risks pertaining to specific drug-event pairs. However, the ROR and PRR methods show high sensitivity but low specificity and are prone to false positives. The MGPS method can pull the disproportionality scores toward the null, thereby reducing the impact of spuriously high ROR values and thus detecting AE signals more reliably. The BCPNN method is a measure of the strength of the drug–AEs association. This integrative approach balances sensitivity and specificity through cross-validation. All analyses were performed in the R (v4.3.2) software.

## 3. Results

### 3.1. Basic Information

The data of crizotinib, ceritinib, alectinib, brigatinib, and loratinib reported in the FAERS database were extracted, spanning the period from the first quarter of 2011 to the fourth quarter of 2023. After excluding duplicates, the number of PS reports for each ALK-TKIs was as follows: crizotinib 19,857, ceritinib 8946, alectinib 12,141, brigatinib 7468, and lorlatinib 8452.

Among patients who reportedly experienced AEs, the majority were female, with most cases involving individuals aged 45–65 years old. The United States was identified as the primary reporting country. In terms of reporters, physicians were reported to be the main source, especially for ceritinib (40.73%) and brigatinib (40.72%), while consumer reports were the highest in alectinib (46.59%). In terms of outcome of AEs, crizotinib (26.35%), ceritinib (28.57%), and lorlatinib (25.04%) were predominantly associated with death as the reported outcome. In contrast, alectinib (22.21%) and brigatinib (26.83%) were mainly linked to hospitalization—initial or prolonged. Notably, most AE reports were submitted within the first 2 months of medication use. For more details, see Table 1 and Figure S1.

### 3.2. Distribution of AE Signals in SOCs

The AEs associated with the five ALK-TKIs were categorized into 21 SOCs according to MedDRA. Disproportionality analysis was performed using the ROR and PRR to identify significant AE signals across multiple SOCs. The analysis revealed distinctive SOCs-level safety profiles for each ALK-TKI, with statistically significant signals summarized below and detailed in Table 2. Crizotinib exhibited prominent signals in neoplasms benign, malignant and unspecified (incl cysts and polyps) (ROR = 3.46, 95% CI = 3.29–3.63, PRR = 3.24, χ^2^ = 2766.34) and eye disorders (ROR = 2.38, 95% CI = 2.23–2.55, PRR = 2.32, χ^2^ = 676.52). Ceritinib demonstrated notable signals for gastrointestinal disorders (ROR = 2.33, 95% CI = 2.20–2.46, PRR = 2.09, χ^2^ = 940.32), neoplasms benign, malignant, and unspecified (incl cysts and polyps) (ROR = 7.22, 95% CI = 6.82–7.64, PRR = 6.14, χ^2^ = 6534.7), metabolism and nutrition disorders (ROR = 2.1, 95% CI = 1.89–2.33, PRR = 2.05, χ^2^ = 200.02), hepatobiliary disorders (ROR = 3.68, 95% CI = 3.25–4.18, PRR = 3.61, χ^2^ = 472.62). Alectinib showed a pronounced signal for hepatobiliary disorders (ROR = 3.30, 95% CI = 2.96–3.69, PRR = 3.24, χ^2^ = 498.68). Brigatinib was predominantly associated with neoplasms benign, malignant, and unspecified (incl cysts and polyps) (ROR = 5.40, 95% CI = 5.07–5.76, PRR = 4.75, χ^2^ = 3374.71). Lorlatinib demonstrated a dominant signal for metabolism and nutrition disorders (ROR = 2.58, 95% CI = 2.34–2.85, PRR = 2.51, χ^2^ = 378.10).

### 3.3. Analysis of AE Signals at the PT Level

The study analyzed AE signals at the PT level using four disproportionality algorithms. After excluding death, malignancy-related AEs, and COVID-19, a total of 454 statistically significant AE signals were identified across the five ALK-TKIs: crizotinib (104 signals), ceritinib (89 signals), alectinib (94 signals), brigatinib (52 signals), and lorlatinib (115 signals). The top 30 AE signals for each ALK-TKI were summarized and compared with those listed in their respective drug labels (Table 3, Table 4, Table 5, Table 6 and Table 7). Key findings are listed below.

Crizotinib: The most frequently reported AEs were vomiting, visual impairment, and constipation. Notably, notable signals were observed for pleural effusion, pericardial effusion and hypoxia, which are not currently mentioned on the drug label. Ceritinib: Diarrhea, nausea, and vomiting were the most commonly reported AEs. Strong but unlisted signals were observed for pleural effusion, elevated C-reactive protein, hypokalemia, and central nervous system lesions. Alectinib: The most frequent AEs were constipation, myalgia, and oedema. Significant signals included pleural effusion, pulmonary oedema, hemolysis, hemolytic anemia, pericardial effusion, fluid retention, and diverticulitis, which are not yet documented in the drug label. Brigatinib: The most prominent AE was blood creatine phosphokinase increased, followed by metastases to the central nervous system. Notable AE signals not mentioned in the label included pleural effusion, hemoptysis, central nervous system lesion, cerebral hemorrhage, pneumonia bacterial, and cerebral edema. Lorlatinib: The most frequently reported AE was blood cholesterol increase, followed by hallucination and weight increase. Significant signals not yet listed in the label included pleural effusion, hemoglobin decreased, low-density lipoprotein increased, pericardial effusion, electrocardiogram QT prolonged, fluid retention, and brain oedema. These findings underscore the importance of tailored monitoring based on the unique AE profiles of each ALK-TKI.

### 3.4. Hepatotoxicity Analysis of ALK-TKIs

A Venn diagram of AE signals was plotted for the five drugs, and all drugs were associated with DILI AEs (Figure S2 and Table S4). A total of 1978 reports of DILI were identified in FAERS, with 696 reports of crizotinib, 451 reports of ceritinib, 528 reports of alectinib, 178 reports of brigatinib, and 125 reports of lorlatinib. Demographic and clinical characteristics of DILI cases associated with ALK-TKIs were analyzed based on the FAERS database. Brigatinib users were generally older (60.74 ± 12.95 years), whereas ceritinib (53.78 ± 17.83) and lorlatinib (53.01 ± 16.30) were reported in younger populations. Females predominated in all groups except for lorlatinib. Geographic distribution indicated that the United States was the primary reporting country for crizotinib (40.23%) and alectinib (30.68%) DILI cases. In terms of reporters, physicians were reported to be the main source, especially for crizotinib (59.63%) and alectinib (57.77%). DILI-related mortality was elevated with crizotinib (16.95%) and lorlatinib (16.00%) but lower for alectinib (4.73%). Most cases lacked medication timing data, though brigatinib had more early-onset reports (≤28 days: 14.05%). These findings highlight the need for tailored monitoring based on drug-specific risk profiles (Table S5).

After assessing the association between each ALK inhibitor and DILI by using the four algorithms, we found that crizotinib, ceritinib, and alectinib were significantly associated with increased DILI reports. Signal analysis was performed for ALK-TKIs-associated DILI. The results showed that the top three reported AEs were alanine aminotransferase (ALT) increased (ROR = 5.65, 95% CI = 4.95–6.47, PRR = 5.63, χ^2^ = 819.84, IC = 2.49, IC025 = 0.82, EBGM = 5.61, EBGM05 = 5.02), aspartate aminotransferase (AST) increased (ROR = 6.52, 95% CI = 5.68–7.48, PRR = 6.49, χ^2^ = 953.29, IC = 2.69, IC025 = 1.03, EBGM = 6.47, EBGM05 = 5.76), and hepatic enzyme increased (ROR = 3.72, 95% CI = 3.2–4.32, PRR = 3.71, χ^2^ = 340.12, IC = 1.89, IC025 = 0.22, EBGM = 3.7, EBGM05 = 3.27) (Table 8 and Table S6).

## 4. Discussion

This large-scale pharmacovigilance study systematically characterized the safety profiles of ALK-TKIs using the FAERS database from 2011 to 2023. Through analyzing 56,864 AE reports and conducting disproportionality analysis, we identified both established and previously unreported safety signals across the five drugs and highlighted significant hepatotoxicity risks associated with crizotinib, ceritinib, and alectinib.

Distinct demographic patterns were observed among patients experiencing AEs. Our demographic analysis revealed that the majority of AE reports were from females, which aligns with the findings of an evaluation of gender differences in adverse drug reactions across various therapeutic interventions, potentially reflecting the influence of sex hormones on transporter proteins, receptors, and enzymes involved in drug metabolism [21,22]. Age distribution further highlighted risks in older populations, concordant with the NSCLC epidemiology and age-related declines in hepatic/renal function affecting drug clearance [23,24]. The geographical analysis demonstrated disparities, with the majority of AE reports originating from the United States and Japan contributing to 58.82% of the total reports. The results were likely influenced by regional variations in drug approval timelines and prescription patterns. Identifying these differences in AEs could reduce the experience of AEs for patients and could be conducive to the development of personalized medicine.

Importantly, our findings on eye disorders, gastrointestinal disorders, hepatobiliary disorders, and metabolism and nutrition disorders are consistent with prior clinical trials and meta-analysis, thus reinforcing the validity of our methodology [12,13,14,15,25]. For instance, crizotinib was identified as the sole agent with a significant signal for ocular toxicity, which aligns with clinical trial data indicating that approximately 62% of patients experienced visual disturbances, such as photopsia, diplopia, and photophobia [21]. Preclinical studies have suggested that crizotinib-induced ocular toxicity may result from the modulation of phototransduction pathways in retinal ganglion cells, as demonstrated in murine models that exhibited altered light sensitivity thresholds [25]. Similarly, the high reporting rate of ceritinib-associated hepatotoxicity observed in our study corresponds with pooled analyses reporting elevated ALT levels in more than 50% of patients [26]. Notably, ceritinib and alectinib both showed elevated hepatobiliary disorder signals, likely attributable to ceritinib’s dual inhibition of insulin and IGF-1 receptors, which can disrupt hepatic metabolic homeostasis [27]. Conversely, lorlatinib demonstrated a more favorable hepatotoxicity profile but showed strong associations with metabolic disturbances, particularly hypercholesterolemia which was a recognized class effect. Emerging evidence has linked these lipid abnormalities to accelerated atherosclerosis, necessitating proactive and vigilant monitoring of lipids profiles in high-risk populations [28]. These findings confirm that disproportionality analysis effectively identifies clinically meaningful signals, and our results align with real-world clinical observations.

In addition to confirming known AEs, our analysis identified several previously unreported AEs that are not currently included in drug labels. Notably, pleural and pericardial effusions emerged as class-wide signals across all ALK-TKIs. While these effusions may reflect progression in NSCLC patients, they could also result from drug-specific mechanisms such as crizotinib-induced endothelial leakage [29]. Among ceritinib-treated patients, hypokalemia has been observed, likely attributable to pharmacodynamic interactions with diuretics commonly prescribed for comorbid conditions [30]. Additionally, our study confirmed a previously underreported association between alectinib and pulmonary edema, corroborating data from the Japanese Adverse Drug Event Report (JADER) database, which documented pulmonary edema in 6 out of 157 cases of alectinib-associated lung toxicity [29]. Furthermore, our study corroborates previous case reports documenting hemolysis and hemolytic anemia as rare but significant AEs associated with alectinib [31]. While no prior studies have reported certain AEs identified in our analysis, where their clinical significance is supported by both high reporting frequencies and robust Bayesian statistical signals, suggesting a potential class effect of ALK-TKIs.

Additionally, this analysis demonstrated consistent hepatotoxicity signals across all five ALK-TKIs, aligning with a meta-analysis reporting elevated hepatotoxicity risks in NSCLC patients treated with ALK inhibitors (pooled incidence: 26% for ALT elevation and 23.2% for AST elevation) [32]. However, the DILI reporting rates in FAERS differed from those observed in registration trials. For instance, ceritinib-associated ALT elevation was reported in 42% of patients (Grade 1–2: 22%) in the ASCEND-5 trial [15], a proportion higher than the FAERS-derived reporting rate. This divergence is likely attributable to the underreporting of asymptomatic laboratory abnormalities in real-world pharmacovigilance systems, where mild or transient elevations may not trigger clinical action or documentation in real-world settings. These findings underscore the complementary roles of clinical trials (systematically capturing graded laboratory abnormalities) and pharmacovigilance databases (detecting severe or unmonitored AEs) in comprehensive risk characterization [12,15,32].

The demographic patterns in DILI reports mirrored NSCLC epidemiology and pharmacovigilance trends. The older age of brigatinib users aligns with age-related declines in hepatic metabolic capacity [24], whereas younger cohorts receiving ceritinib and lorlatinib may reflect preferential prescribing patterns for these agents in non-smokers with ALK rearrangements [6]. Female predominance in DILI reports parallels gender disparities in NSCLC incidence and AE reporting behaviors observed in pharmacovigilance studies [22]. Geographic clustering, such as U.S.-dominant crizotinib reports, correlates with regional variations in drug approval timelines and clinical adoption rates [10].

Disproportionality analysis confirmed robust hepatotoxicity signals for crizotinib, ceritinib, and alectinib, corroborating previous clinical trial evidence while advancing real-world evidence through quantitative risk ranking [13,14,15,33]. Ceritinib exhibited particularly elevated hepatotoxicity risk, potentially attributable to its broad-spectrum kinase inhibition profile that may impair hepatic regenerative mechanisms. Conversely, brigatinib and lorlatinib demonstrated more favorable hepatic safety profiles, suggesting their potential suitability for patients with pre-existing liver dysfunction. Nevertheless, the precise mechanisms underlying ALK-TKIs-associated hepatotoxicity remain incompletely understood, highlighting the need for further research to elucidate these pathways.

The clinical implications of our findings are significant, as they provide actionable insights for guiding the selection and monitoring of ALK-TKIs. The identification of previously unreported AEs underscores the need for heightened vigilance among healthcare providers, particularly when prescribing these agents to manage patients with comorbid conditions or predisposing risk factors. Prior to initiating ALK-TKI therapy, clinicians should conduct comprehensive baseline assessments to tailor ALK-TKI selection based on individual patient comorbidities. For instance, lorlatinib should be avoided in patients with pre-existing hyperlipidemia due to its propensity to exacerbate lipid abnormalities. Proactive surveillance and early intervention are vital during therapy, so as to mitigate AE progression and prevent severe complications. In addition, this study systematically compiled the currently labeled warnings, recommended dose adjustments for hepatic impairment, and liver monitoring guidelines for each ALK-TKI (Table S7). By comparing labeled warnings, dose adjustment protocols, and monitoring requirements across ALK-TKIs, we delineated distinct hepatotoxicity risk profiles and management strategies among these agents. These findings, integrated with real-world evidence from the FAERS database, provide actionable insights to guide personalized treatment selection based on individual patient profiles, particularly hepatic function status. By integrating personalized treatment strategies with rigorous AE monitoring, the risk-benefit profile of ALK-TKIs can be optimized for individual patients [26].

The limitations of the study should be considered when interpreting the results. Firstly, the inherent constraints of the FAERS database, as a spontaneous reporting system, introduce potential biases such as underreporting, selective reporting, and confounding due to underlying disease progression. Secondly, disproportionality metrics indicate association rather than causation. While our study established a statistically significant association between ALK-TKIs and DILI within the FAERS database, these findings do not imply causation. To validate these findings clinically, further research is warranted across three key domains: (1) Prospective cohort studies to assess causality and quantify incidence rates in real-world populations through longitudinal monitoring of ALK-TKI recipients; (2) pharmacogenomic analyses leveraging next-generation sequencing to identify genetic predispositions (e.g., HLA haplotypes, CYP450 polymorphisms) associated with specific AEs; (3) mechanistic investigations integrating in vitro models (e.g., patient-derived organoids), multi-omics profiling, and biomarker discovery to elucidate the pathophysiological pathways underlying pericardial effusion or hemolytic anemia. Findings from such investigations will help refine risk stratification and aid the development of targeted preventive measures. Additionally, the Weber effect may lead to inflated AE reporting rates for newer drugs. To contextualize the potential impact of the Weber effect, this study compiled the first approval dates of each ALK-TKI in the United States, European Union, Japan, and China, as well as in other countries (Table S8). While this study did not apply statistical adjustments (e.g., time-weighted models) to mitigate the Weber effect—potentially resulting in overestimated safety signals for newer drugs—the inclusion of approval timelines provides critical context for interpreting these findings. Specifically, readers are cautioned against overinterpreting safety signals for recently approved agents (e.g., lorlatinib, approved in 2018). Future studies should incorporate time-stratified analyses or advanced statistical approaches (e.g., negative binomial regression with temporal weighting) to account for reporting biases associated with the Weber effect.

## 5. Conclusions

In conclusion, this large-scale pharmacovigilance database analysis comprehensively analyzed AE profiles of five ALK-TKIs used in NSCLC, revealing both established and previously unrecognized safety signals. In clinical settings, these signals need to be monitored and product labels may be updated to inform patients and clinicians of the AEs. Meanwhile, as noted in the reports, the five ALK-TKIs examined in this study were strongly associated with DILI. The study established a hepatotoxicity gradient among ALK-TKIs, thereby guiding agent selection for patients with hepatic comorbidities. Further studies are required to comprehend the mechanisms underlying these unlabeled AEs and to establish a causal relationship between the drugs and DILI, thus supporting the development of standardized monitoring guidelines.

## Figures and Tables

**Table 1:** Basic information for crizotinib, ceritinib, alectinib, brigatinib, and lorlatinib.

Characteristics	Crizotinib	Ceritinib	Alectinib	Brigatinib	Lorlatinib
**Total number**	19,857	8946	12,141	7468	8452
**Age (years), mean ± SD**	60.13 ± 16.56	55.84 ± 14.96	60.23 ± 14.72	61.03 ± 14.93	55.84 ± 17.96
**Sex**					
Female	10,748 (54.13)	4639 (51.86)	6977 (57.47)	4221 (56.52)	4370 (51.70)
Male	7770 (39.13)	3646 (40.76)	4235 (34.88)	2750 (36.82)	3306 (39.12)
Unknown	1339 (6.74)	661 (7.39)	929 (7.65)	497 (6.66)	776 (9.18)
**Reporter country**					
United states	11,814 (59.50)	1832 (20.48)	6749 (55.59)	3984 (53.35)	2695 (31.89)
Japan	1670 (8.41)	1357 (15.17)	974 (8.02)	1294 (17.33)	1079 (12.77)
China	527 (2.65)	638 (7.13)	572 (4.71)	48 (0.64)	269 (3.18)
Other	5846 (29.44)	5119 (57.22)	3846 (31.68)	2142 (28.68)	4409 (52.17)
**Reporter**					
Physician	7450 (37.52)	3644 (40.73)	4193 (34.54)	3041 (40.72)	3253 (38.49)
Consumer	5587 (28.14)	3037 (33.95)	5657 (46.59)	2629 (35.20)	3377 (39.96)
Pharmacist	2332 (11.74)	350 (3.91)	951 (7.83)	521 (6.89)	563 (6.66)
Other health professionals	4394 (22.13)	1491 (16.67)	1238 (10.20)	1272 (17.03)	1136 (13.44)
Unknown	94 (0.47)	424 (4.74)	102 (0.84)	5 (0.07)	123 (1.46)
**Outcome**					
Death	5232 (26.35)	2556 (28.57)	1336 (11.00)	1379 (18.47)	2116 (25.04)
Hospitalization—initial or prolonged	4200 (21.15)	2152 (24.06)	2697 (22.21)	2004 (26.83)	1784 (21.11)
Life threatening	348 (1.75)	242 (2.71)	236 (1.94)	72 (0.96)	142 (1.68)
Disability	86 (0.43)	87 (0.97)	128 (1.05)	34 (0.46)	93 (1.10)
Other serious outcomes	3896 (19.62)	3067 (34.28)	4488 (36.97)	3183 (42.62)	3120 (36.91)
Unknown	6095 (30.69)	842 (9.41)	3256 (26.82)	796 (10.66)	1197 (14.16)
**Adverse event occurrence time-medication date (days)**					
0–7	1570 (7.91)	1294 (14.46)	111 (0.91)	531 (7.11)	222 (2.63)
7–28	1600 (8.06)	1179 (13.18)	351 (2.89)	330 (4.42)	408 (4.83)
28–60	857 (4.32)	642 (7.18)	249 (2.05)	153 (2.05)	352 (4.16)
≥60	2072 (10.43)	1707 (19.08)	452 (3.72)	470 (6.29)	738 (8.73)
Unknown	13,758 (69.29)	4124 (46.10)	9612 (79.17)	5984 (80.13)	6448 (76.29)

SD standard deviation.

**Table 2:** Signal strength at the SOC level of AEs for ALK-TKIs.

Drug	SOC	N	ROR (95% CI)	PRR (χ^2^)	IC (IC025)	EBGM (EBGMO5)
crizotinib	neoplasms benign, malignant, and unspecified (incl cysts and polyps)	1738	3.46 (3.29–3.63) ^★^	3.24 (2766.34) ^★^	1.70 (0.03) ^★^	3.24 (3.11) ^★^
	eye disorders	883	2.38 (2.23–2.55) ^★^	2.32 (676.52) ^★^	1.21 (−0.45)	2.32 (2.19) ^★^
ceritinib	gastrointestinal disorders	1557	2.33 (2.2–2.46) ^★^	2.09 (940.32) ^★^	1.06 (−0.60)	2.09 (2.00) ^★^
	neoplasms benign, malignant, and unspecified (incl cysts and polyps)	1543	7.22 (6.82–7.64) ^★^	6.14 (6534.7) ^★^	2.62 (0.95) ^★^	6.14 (5.86) ^★^
	metabolism and nutrition disorders	369	2.1 (1.89–2.33) ^★^	2.05 (200.02) ^★^	1.04 (−0.63)	2.05 (1.88)
	hepatobiliary disorders	252	3.68 (3.25–4.18) ^★^	3.61 (472.62) ^★^	1.85 (0.18) ^★^	3.60 (3.24) ^★^
alectinib	hepatobiliary disorders	319	3.30 (2.96–3.69) ^★^	3.24 (498.68) ^★^	1.70 (0.03) ^★^	3.24 (2.95) ^★^
brigatinib	neoplasms benign, malignant, and unspecified (incl cysts and polyps)	1106	5.40 (5.07–5.76) ^★^	4.75 (3374.71) ^★^	2.25 (0.58) ^★^	4.74 (4.5) ^★^
lorlatinib	metabolism and nutrition disorders	410	2.58 (2.34–2.85) ^★^	2.51 (378.10) ^★^	1.32 (−0.34)	2.50 (2.31) ^★^

^★^ indicates statistical significance. SOC, System Organ Class in MedDRA; ROR, Reporting Odds Ratio; PRR, Proportional Reporting Ratio; IC, Information Component; EBGM, Empirical Bayesian Geometric Mean.

**Table 3:** The top 30 signals in the AE reports of crizotinib.

PT	N	ROR (95% CI)	PRR (χ^2^)	IC (IC025)	EBGM (EBGMO5)
vomiting	448	3.25 (2.96–3.57)	3.20 (683.06)	1.68 (0.01)	3.20 (2.96)
visual impairment	292	7.30 (6.51–8.20)	7.21 (1561.41)	2.85 (1.18)	7.20 (6.53)
constipation	283	4.11 (3.66–4.63)	4.07 (656.68)	2.02 (0.36)	4.07 (3.68)
oedema peripheral	262	7.47 (6.61–8.44)	7.38 (1444.58)	2.88 (1.21)	7.37 (6.65)
pleural effusion ^a^	157	8.23 (7.03–9.63)	8.17 (986.21)	3.03 (1.36)	8.15 (7.15)
dysgeusia	153	7.04 (6.00–8.25)	6.99 (784.19)	2.80 (1.14)	6.97 (6.10)
dysphagia	125	4.38 (3.67–5.22)	4.36 (323.69)	2.12 (0.46)	4.36 (3.76)
interstitial lung disease	118	7.75 (6.46–9.29)	7.71 (687.39)	2.94 (1.28)	7.69 (6.61)
oedema	116	6.84 (5.70–8.22)	6.81 (574.13)	2.76 (1.10)	6.80 (5.83)
photopsia	115	61.48 (51.08–73.99)	61.13 (6656.83)	5.90 (4.24)	59.84 (51.25)
pulmonary embolism	109	3.66 (3.03–4.42)	3.64 (209.18)	1.86 (0.20)	3.64 (3.11)
renal impairment	100	3.62 (2.98–4.41)	3.61 (188.57)	1.85 (0.18)	3.61 (3.06)
pneumonitis	94	11.35 (9.26–13.9)	11.30 (879.35)	3.49 (1.83)	11.26 (9.50)
hepatic enzyme increase	88	4.21 (3.42–5.20)	4.20 (214.47)	2.07 (0.40)	4.20 (3.52)
alanine aminotransferase increase	84	4.85 (3.92–6.02)	4.84 (255.5)	2.27 (0.61)	4.83 (4.04)
blood creatinine increased	75	3.69 (2.94–4.64)	3.68 (146.67)	1.88 (0.21)	3.68 (3.04)
respiratory failure	72	3.43 (2.72–4.33)	3.43 (123.65)	1.78 (0.11)	3.42 (2.82)
aspartate aminotransferase increase	71	4.95 (3.92–6.26)	4.94 (222.94)	2.30 (0.64)	4.93 (4.06)
bradycardia	70	4.52 (3.57–5.72)	4.51 (190.99)	2.17 (0.50)	4.50 (3.70)
vitreous floaters	63	20.72 (16.16–26.55)	20.65 (1169.71)	4.36 (2.69)	20.51 (16.66)
oesophagitis	60	19.01 (14.74–24.51)	18.95 (1013.72)	4.24 (2.57)	18.83 (15.22)
pulmonary oedema	59	4.34 (3.36–5.61)	4.33 (151.21)	2.11 (0.45)	4.33 (3.50)
hepatic function abnormal	57	5.08 (3.92–6.59)	5.07 (185.97)	2.34 (0.67)	5.06 (4.07)
liver function test abnormal	55	6.85 (5.26–8.93)	6.83 (273.36)	2.77 (1.10)	6.82 (5.46)
pericardial effusion ^a^	54	7.69 (5.88–10.04)	7.67 (312.4)	2.94 (1.27)	7.65 (6.12)
electrocardiogram qt prolonged	52	4.77 (3.63–6.26)	4.76 (154.21)	2.25 (0.58)	4.75 (3.78)
transaminases increased	44	6.45 (4.80–8.68)	6.44 (201.88)	2.68 (1.02)	6.43 (5.02)
hypoxia ^a^	42	4.03 (2.97–5.45)	4.02 (95.18)	2.01 (0.34)	4.02 (3.12)
liver function test increased	39	5.45 (3.98–7.46)	5.44 (141.12)	2.44 (0.78)	5.43 (4.18)
diplopia	37	4.87 (3.53–6.73)	4.86 (113.38)	2.28 (0.61)	4.86 (3.71)

^a^ indicates a PT not yet mentioned in the drug instruction. PT, Preferred Term in MedDRA; ROR, Reporting Odds Ratio; PRR, Proportional Reporting Ratio; IC, Information Component; EBGM, Empirical Bayesian Geometric Mean.

**Table 4:** The top 30 signals in the AE reports of ceritinib.

PT	N	ROR (95% CI)	PRR (χ^2^)	IC (IC025)	EBGM (EBGMO5)
diarrhea	422	4.54 (4.12–5.01)	4.37 (1108.54)	2.13 (0.46)	4.37 (4.03)
nausea	350	3.30 (2.97–3.68)	3.21 (539.71)	1.68 (0.02)	3.21 (2.94)
vomiting	254	4.20 (3.71–4.76)	4.11 (600.80)	2.04 (0.37)	4.10 (3.70)
metastases to central nervous system	173	107.08 (91.99–124.65)	105.03 (17,486.21)	6.69 (5.02)	103.03 (90.73)
decreased appetite	126	3.63 (3.05–4.33)	3.60 (236.80)	1.85 (0.18)	3.59 (3.10)
alanine aminotransferase increase	72	9.70 (7.69–12.24)	9.63 (556.55)	3.27 (1.60)	9.62 (7.92)
aspartate aminotransferase increase	64	10.51 (8.22–13.45)	10.44 (545.90)	3.38 (1.72)	10.43 (8.49)
blood creatinine increase	64	7.25 (5.67–9.28)	7.21 (342.15)	2.85 (1.18)	7.20 (5.86)
general physical health deterioration	60	3.90 (3.02–5.02)	3.88 (128.29)	1.95 (0.29)	3.88 (3.13)
hepatic function abnormal	56	11.28 (8.67–14.68)	11.22 (520.37)	3.48 (1.82)	11.20 (8.98)
metastases to liver	55	21.81 (16.72–28.44)	21.68 (1080.78)	4.43 (2.77)	21.59 (17.29)
pleural effusion ^a^	52	6.37 (4.85–8.37)	6.34 (233.88)	2.66 (1.00)	6.33 (5.04)
pericardial effusion	47	15.36 (11.53–20.47)	15.28 (625.89)	3.93 (2.26)	15.24 (11.99)
pericarditis	46	19.79 (14.81–26.46)	19.70 (813.58)	4.29 (2.63)	19.63 (15.40)
renal impairment	45	3.59 (2.67–4.81)	3.57 (83.42)	1.84 (0.17)	3.57 (2.79)
metastases to bone	44	19.20 (14.27–25.83)	19.11 (752.59)	4.25 (2.58)	19.04 (14.86)
blood alkaline phosphatase increase	41	14.71 (10.82–20.00)	14.65 (520.11)	3.87 (2.20)	14.61 (11.30)
hepatic enzyme increase	40	4.17 (3.06–5.69)	4.15 (95.85)	2.05 (0.39)	4.15 (3.20)
liver disorder	39	6.54 (4.78–8.96)	6.52 (182.15)	2.70 (1.04)	6.51 (5.01)
hyperglycemia	38	8.29 (6.03–11.40)	8.26 (242.23)	3.04 (1.38)	8.25 (6.32)
pneumonitis	31	7.88 (5.54–11.22)	7.86 (185.42)	2.97 (1.31)	7.85 (5.84)
gamma-glutamyltransferase increase	30	11.19 (7.81–16.02)	11.15 (276.82)	3.48 (1.81)	11.13 (8.25)
metastases to meninges	28	95.64 (65.78–139.06)	95.35 (2568.40)	6.55 (4.88)	93.70 (68.51)
transaminases increase	28	9.07 (6.26–13.15)	9.05 (200.14)	3.18 (1.51)	9.03 (6.62)
c-reactive protein increase ^a^	26	4.35 (2.96–6.40)	4.34 (66.89)	2.12 (0.45)	4.34 (3.14)
drug resistance	26	6.75 (4.59–9.93)	6.74 (126.91)	2.75 (1.08)	6.73 (4.88)
hypokalemia ^a^	26	4.21 (2.86–6.18)	4.20 (63.29)	2.07 (0.40)	4.19 (3.04)
central nervous system lesion ^a^	24	11.03 (7.39–16.47)	11.00 (217.85)	3.46 (1.79)	10.98 (7.85)
electrocardiogram qt prolonged	23	4.64 (3.08–6.99)	4.63 (65.52)	2.21 (0.54)	4.63 (3.29)
gastrointestinal toxicity	21	34.22 (22.27–52.58)	34.14 (671.29)	5.08 (3.42)	33.93 (23.68)

^a^ indicates a PT not yet mentioned in the drug instruction. PT, Preferred Term in MedDRA; ROR, Reporting Odds Ratio; PRR, Proportional Reporting Ratio; IC, Information Component; EBGM, Empirical Bayesian Geometric Mean.

**Table 5:** The top 30 signals in the AE reports of alectinib.

PT	N	ROR (95% CI)	PRR (χ^2^)	IC (IC025)	EBGM (EBGMO5)
constipation	285	6.71 (5.96–7.55)	6.57 (1349.54)	2.71 (1.05)	6.56 (5.95)
myalgia	159	5.24 (4.48–6.13)	5.19 (538.19)	2.37 (0.71)	5.18 (4.55)
oedema	95	10.38 (8.48–12.71)	10.31 (796.76)	3.36 (1.70)	10.28 (8.68)
oedema peripheral	93	5.47 (4.46–6.71)	5.44 (336.85)	2.44 (0.78)	5.43 (4.58)
metastases to central nervous system	89	39.37 (31.92–48.55)	39.09 (3267.58)	5.27 (3.61)	38.67 (32.45)
pleural effusion ^a^	80	7.44 (5.97–9.27)	7.40 (441.96)	2.88 (1.22)	7.38 (6.14)
pneumonitis	75	13.68 (10.89–17.17)	13.60 (872.46)	3.76 (2.09)	13.55 (11.20)
blood bilirubin increase	73	17.10 (13.58–21.54)	17.01 (1094.85)	4.08 (2.42)	16.93 (13.96)
bradycardia	73	7.81 (6.21–9.84)	7.77 (430.16)	2.96 (1.29)	7.76 (6.40)
blood creatine phosphokinase increase	69	17.42 (13.74–22.08)	17.33 (1056.70)	4.11 (2.44)	17.25 (14.14)
blood creatinine increase	57	4.76 (3.67–6.18)	4.74 (168.25)	2.24 (0.58)	4.74 (3.81)
pulmonary oedema ^a^	57	7.30 (5.62–9.47)	7.27 (307.74)	2.86 (1.19)	7.26 (5.84)
hepatic function abnormal	53	7.73 (5.90–10.13)	7.70 (308.70)	2.94 (1.28)	7.69 (6.13)
interstitial lung disease	51	5.44 (4.13–7.16)	5.42 (183.65)	2.44 (0.77)	5.41 (4.30)
aspartate aminotransferase increase	50	6.08 (4.60–8.03)	6.06 (210.88)	2.60 (0.93)	6.05 (4.79)
hepatic enzyme increase	50	3.78 (2.86–4.99)	3.77 (101.69)	1.91 (0.25)	3.77 (2.98)
hemolysis ^a^	49	37.11 (27.99–49.20)	36.96 (1697.01)	5.19 (3.53)	36.59 (28.90)
photosensitivity reaction	49	14.85 (11.21–19.68)	14.80 (627.98)	3.88 (2.22)	14.74 (11.65)
hemolytic anemia ^a^	43	31.55 (23.35–42.62)	31.44 (1256.36)	4.96 (3.30)	31.17 (24.24)
pericardial effusion ^a^	43	10.51 (7.79–14.18)	10.47 (367.53)	3.38 (1.72)	10.45 (8.13)
fluid retention ^a^	41	3.94 (2.90–5.35)	3.93 (89.52)	1.97 (0.31)	3.93 (3.04)
liver disorder	41	5.05 (3.71–6.86)	5.04 (132.48)	2.33 (0.66)	5.03 (3.89)
alanine aminotransferase increase	39	3.86 (2.82–5.29)	3.85 (82.25)	1.94 (0.28)	3.85 (2.96)
lung disorder	37	3.78 (2.74–5.22)	3.77 (75.35)	1.91 (0.25)	3.77 (2.88)
liver function test increase	31	5.50 (3.86–7.82)	5.48 (113.54)	2.45 (0.79)	5.48 (4.08)
drug resistance	29	5.33 (3.70–7.67)	5.32 (101.58)	2.41 (0.74)	5.31 (3.92)
hyperbilirubinemia	28	15.56 (10.73–22.57)	15.53 (379.02)	3.95 (2.28)	15.47 (11.33)
transaminases increase	27	6.45 (4.42–9.41)	6.43 (123.76)	2.68 (1.02)	6.43 (4.68)
metastases to bone	26	8.30 (5.65–12.20)	8.29 (166.24)	3.05 (1.38)	8.27 (5.99)
diverticulitis ^a^	24	3.80 (2.54–5.67)	3.79 (49.35)	1.92 (0.26)	3.79 (2.71)

^a^ indicates a PT not yet mentioned in the drug instruction. PT, Preferred Term in MedDRA; ROR, Reporting Odds Ratio; PRR, Proportional Reporting Ratio; IC, Information Component; EBGM, Empirical Bayesian Geometric Mean.

**Table 6:** The top 30 signals in the AE reports of brigatinib.

PT	N	ROR (95% CI)	PRR (χ^2^)	IC (IC025)	EBGM (EBGMO5)
blood creatine phosphokinase increase	141	59.73 (50.51–70.63)	58.62 (7895.64)	5.86 (4.19)	57.95 (50.37)
metastases to central nervous system	141	102.48 (86.61–121.26)	100.56 (13,627.45)	6.62 (4.96)	98.60 (85.65)
blood pressure increase	71	3.26 (2.58–4.12)	3.24 (110.05)	1.69 (0.03)	3.24 (2.66)
pleural effusion ^a^	46	7.10 (5.31–9.49)	7.06 (239.23)	2.82 (1.15)	7.05 (5.53)
pneumonitis	40	11.63 (8.52–15.87)	11.57 (385.56)	3.53 (1.86)	11.55 (8.90)
amylase increase	39	85.67 (62.37–117.66)	85.22 (3191.92)	6.39 (4.72)	83.81 (64.27)
renal impairment	37	3.37 (2.44–4.66)	3.36 (61.47)	1.75 (0.08)	3.36 (2.56)
lipase increase	31	42.03 (29.49–59.90)	41.86 (1226.26)	5.38 (3.71)	41.52 (30.87)
aspartate aminotransferase increase	30	5.91 (4.12–8.45)	5.89 (121.60)	2.56 (0.89)	5.88 (4.35)
lung disorder	29	4.79 (3.33–6.90)	4.78 (86.63)	2.26 (0.59)	4.77 (3.52)
metastases to liver	29	13.91 (9.65–20.04)	13.86 (345.08)	3.79 (2.12)	13.82 (10.18)
interstitial lung disease	28	4.79 (3.30–6.94)	4.77 (83.45)	2.25 (0.59)	4.77 (3.49)
photosensitivity reaction	28	14.01 (9.66–20.31)	13.96 (336.03)	3.80 (2.13)	13.92 (10.20)
pulmonary toxicity	26	29.05 (19.74–42.74)	28.95 (697.69)	4.85 (3.18)	28.79 (20.84)
alanine aminotransferase increase	24	3.85 (2.58–5.74)	3.84 (50.37)	1.94 (0.27)	3.84 (2.74)
hemoptysis ^a^	20	6.24 (4.02–9.68)	6.22 (87.62)	2.64 (0.97)	6.22 (4.31)
hepatic function abnormal	20	4.65 (3.00–7.22)	4.64 (57.14)	2.21 (0.55)	4.64 (3.21)
pericardial effusion ^a^	19	7.53 (4.80–11.81)	7.51 (107.08)	2.91 (1.24)	7.50 (5.14)
metastases to bone	16	8.04 (4.92–13.13)	8.02 (98.24)	3.00 (1.34)	8.01 (5.31)
central nervous system lesion ^a^	14	9.24 (5.47–15.61)	9.22 (102.46)	3.20 (1.54)	9.21 (5.93)
hepatotoxicity	14	4.91 (2.91–8.30)	4.91 (43.52)	2.29 (0.63)	4.90 (3.16)
liver function test increase	14	3.89 (2.30–6.58)	3.89 (30.01)	1.96 (0.29)	3.88 (2.50)
hyperglycemia	13	3.51 (2.04–6.05)	3.51 (23.31)	1.81 (0.14)	3.51 (2.22)
cerebral hemorrhage ^a^	12	3.28 (1.86–5.77)	3.27 (18.92)	1.71 (0.04)	3.27 (2.04)
metastases to lung	12	9.03 (5.12–15.91)	9.01 (85.36)	3.17 (1.50)	9.00 (5.60)
taste disorder	12	3.58 (2.03–6.32)	3.58 (22.31)	1.84 (0.17)	3.58 (2.23)
pneumonia bacterial ^a^	11	9.01 (4.98–16.28)	9.00 (78.05)	3.17 (1.50)	8.98 (5.47)
brain oedema ^a^	10	7.74 (4.16–14.40)	7.73 (58.55)	2.95 (1.28)	7.72 (4.59)
metastases to meninges	10	38.58 (20.70–71.91)	38.53 (362.79)	5.26 (3.59)	38.24 (22.72)
hypercholesterolemia	7	8.61 (4.10–18.08)	8.60 (46.96)	3.10 (1.44)	8.59 (4.62)

^a^ indicates a PT not yet mentioned in the drug instruction. PT, Preferred Term in MedDRA; ROR, Reporting Odds Ratio; PRR, Proportional Reporting Ratio; IC, Information Component; EBGM, Empirical Bayesian Geometric Mean.

**Table 7:** The top 30 signals in the AE reports of lorlatinib.

PT	N	ROR (95% CI)	PRR (χ^2^)	IC (IC025)	EBGM (EBGMO5)
blood cholesterol increase	128	23.81 (19.99–28.37)	23.47 (2737.05)	4.54 (2.88)	23.32 (20.14)
hallucination	111	12.10 (10.03–14.60)	11.95 (1111.76)	3.58 (1.91)	11.92 (10.19)
weight increase	109	3.46 (2.86–4.18)	3.43 (187.72)	1.78 (0.11)	3.42 (2.92)
oedema peripheral	103	9.21 (7.58–11.19)	9.11 (743.13)	3.18 (1.52)	9.09 (7.73)
oedema	96	16.03 (13.11–19.62)	15.86 (1332.06)	3.98 (2.32)	15.80 (13.35)
hypercholesterolemia	92	100.00 (81.20–123.15)	98.92 (8680.41)	6.59 (4.92)	96.31 (80.91)
cognitive disorder	90	14.19 (11.52–17.47)	14.05 (1087.52)	3.81 (2.14)	14.00 (11.76)
pleural effusion ^a^	70	10.02 (7.92–12.68)	9.94 (562.02)	3.31 (1.64)	9.92 (8.14)
blood triglycerides increase	66	45.30 (35.05–57.80)	44.95 (2801.84)	5.47 (3.81)	44.41 (36.22)
hyperlipidemia	66	75.75 (59.31–96.75)	75.17 (4731.61)	6.20 (4.54)	73.65 (60.01)
neuropathy peripheral	59	4.35 (3.37–5.62)	4.33 (151.10)	2.11 (0.45)	4.32 (3.49)
hypertriglyceridemia	55	80.17 (61.32–104.81)	79.65 (4179.53)	6.28 (4.62)	77.95 (62.29)
pulmonary embolism	45	5.09 (3.79–6.82)	5.06 (146.69)	2.34 (0.67)	5.06 (3.96)
speech disorder	44	6.93 (5.15–9.32)	6.90 (221.56)	2.78 (1.12)	6.89 (5.37)
hemoglobin decrease ^a^	43	3.20 (2.37–4.32)	3.19 (64.82)	1.67 (0.01)	3.19 (2.48)
nervous system disorder	42	17.73 (13.08–24.03)	17.65 (656.56)	4.13 (2.47)	17.57 (13.62)
low-density lipoprotein increase ^a^	39	46.76 (34.07–64.18)	46.55 (1716.39)	5.52 (3.86)	45.97 (35.27)
cardiac failure	34	3.39 (2.42–4.74)	3.38 (56.91)	1.75 (0.09)	3.38 (2.55)
hallucination, auditory	34	20.17 (14.39–28.27)	20.09 (613.54)	4.32 (2.65)	19.99 (15.07)
pericardial effusion ^a^	34	12.10 (8.64–16.96)	12.06 (343.71)	3.59 (1.92)	12.02 (9.06)
psychotic disorder	33	11.73 (8.33–16.53)	11.69 (321.75)	3.54 (1.88)	11.66 (8.75)
mental disorder	32	5.53 (3.91–7.83)	5.51 (118.09)	2.46 (0.79)	5.51 (4.12)
hallucination, visual	30	11.32 (7.91–16.21)	11.29 (280.47)	3.49 (1.83)	11.25 (8.33)
dyslipidemia	29	41.16 (28.53–59.39)	41.03 (1119.78)	5.34 (3.68)	40.57 (29.85)
pneumonitis	29	7.42 (5.15–10.69)	7.40 (160.19)	2.88 (1.22)	7.38 (5.44)
amnesia	27	3.80 (2.61–5.55)	3.79 (55.51)	1.92 (0.26)	3.79 (2.76)
electrocardiogram qt prolonged ^a^	26	5.55 (3.78–8.16)	5.54 (96.64)	2.47 (0.80)	5.53 (4.01)
delirium	25	6.20 (4.18–9.18)	6.18 (108.42)	2.63 (0.96)	6.17 (4.44)
fluid retention ^a^	24	3.59 (2.41–5.36)	3.59 (44.73)	1.84 (0.17)	3.58 (2.56)
brain oedema ^a^	21	14.76 (9.61–22.66)	14.72 (267.52)	3.87 (2.21)	14.67 (10.24)

^a^ indicates a PT not yet mentioned in the drug instruction. PT, Preferred Term in MedDRA; ROR, Reporting Odds Ratio; PRR, Proportional Reporting Ratio; IC, Information Component; EBGM, Empirical Bayesian Geometric Mean.

**Table 8:** Signal strength of five drug-related DILI.

DRUG	N	DILI reports	DILI Proportion (%)	ROR (95% CI)	PRR (χ^2^)	IC (IC025)	EBGM (EBGMO5)
crizotinib	19,857	696	3.51	3.54 (3.28–3.82) ^★^	3.45 (1220.66) ^★^	1.78 (0.12) ^★^	3.45 (3.23) ^★^
ceritinib	8946	451	5.04	5.25 (4.77–5.77) ^★^	5.03 (1470.92) ^★^	2.33 (0.66) ^★^	5.03 (4.65) ^★^
alectinib	12,141	528	4.35	4.49 (4.12–4.90) ^★^	4.34 (1369.30) ^★^	2.12 (0.45) ^★^	4.34 (4.03) ^★^
brigatinib	7468	178	2.38	2.40 (2.07–2.78) ^★^	2.37 (141.76) ^★^	1.24 (−0.42)	2.37 (2.09) ^★^
lorlatinib	8452	125	1.48	1.49 (1.25–1.77) ^★^	1.48 (19.61)	0.56 (−1.10)	1.48 (1.28)

^★^ indicates statistical significance. ROR, Reporting Odds Ratio; PRR, Proportional Reporting Ratio; IC, Information Component; EBGM, Empirical Bayesian Geometric Mean.

## Data Availability

Data supporting reported results can be made available upon request.

## Supplementary Materials

The following supporting information can be downloaded at: https://www.mdpi.com/article/10.3390/toxics13030210/s1, Figure S1: Distribution of age(a), weight(b), sex(c), country of reporter(d), the type of reporter(e), and outcome(f) in reports for five drugs; Figure S2: Wayne plots of adverse event signals for the five drugs; Table S1: Dictionary of preferred terms for liver injury; Table S2: Contingency table; Table S3: ROR, PRR, BCPNN, and EBGM methods, formulas, and thresholds; Table S4: Common adverse events signals for five drugs; Table S5: Basic information for DILI of crizotinib, ceritinib, alectinib, brigatinib, lorlatinib; Table S6: Signal analysis of DILI in ALK-TKIs; Table S7: Label warnings and hepatic safety guidelines for ALK-TKIs; Table S8: Global approval timeline of ALK-TKIs.

## References

[1] Thai A.A., Solomon B.J., Sequist L.V., Gainor J.F., Heist R.S. (2021). Lung cancer. Lancet.

[2] Travis W.D., Brambilla E., Nicholson A.G., Yatabe Y., Austin J.H.M., Beasley M.B., Chirieac L.R., Dacic S., Duhig E., Flieder D.B. (2015). The 2015 World Health Organization Classification of Lung Tumors. J. Thorac. Oncol..

[3] Remon J., Soria J.C., Peters S. (2021). Early and locally advanced non-small-cell lung cancer: An update of the ESMO Clinical Practice Guidelines focusing on diagnosis, staging, systemic and local therapy. Ann. Oncol..

[4] Skribek M., Rounis K., Tsakonas G., Ekman S. (2022). Complications following novel therapies for non-small cell lung cancer. J. Intern. Med..

[5] Soda M., Choi Y.L., Enomoto M., Takada S., Yamashita Y., Ishikawa S., Fujiwara S., Watanabe H., Kurashina K., Hatanaka H. (2007). Identification of the transforming EML4-ALK fusion gene in non-small-cell lung cancer. Nature.

[6] Shaw A.T., Engelman J.A. (2013). ALK in lung cancer: Past, present, and future. J. Clin. Oncol..

[7] Solomon B., Varella-Garcia M., Camidge D.R. (2009). ALK gene rearrangements: A new therapeutic target in a molecularly defined subset of non-small cell lung cancer. J. Thorac. Oncol..

[8] Wang L., Wang W. (2020). Safety and efficacy of anaplastic lymphoma kinase tyrosine kinase inhibitors in non-small cell lung cancer (Review). Oncol. Rep..

[9] Ettinger D.S., Wood D.E., Akerley W., Bazhenova L.A., Borghaei H., Camidge D.R., Cheney R.T., Chirieac L.R., D’Amico T.A., Demmy T.L. (2015). Non-Small Cell Lung Cancer, Version 6.2015. J. Natl. Compr. Cancer Netw..

[10] Omar N.E., Fahmy Soliman A.I., Eshra M., Saeed T., Hamad A., Abou-Ali A. (2021). Postmarketing safety of anaplastic lymphoma kinase (ALK) inhibitors: An analysis of the FDA Adverse Event Reporting System (FAERS). ESMO Open.

[11] Kumachev A., Wu P.E. (2021). Drug-induced liver injury. Can. Med. Assoc. J..

[12] Huber R.M., Hansen K.H., Paz-Ares Rodríguez L., West H.L., Reckamp K.L., Leighl N.B., Tiseo M., Smit E.F., Kim D.W., Gettinger S.N. (2020). Brigatinib in Crizotinib-Refractory ALK+ NSCLC: 2-Year Follow-up on Systemic and Intracranial Outcomes in the Phase 2 ALTA Trial. J. Thorac. Oncol..

[13] Michels S., Massutí B., Schildhaus H.U., Franklin J., Sebastian M., Felip E., Grohé C., Rodriguez-Abreu D., Abdulla D.S.Y., Bischoff H. (2019). Safety and Efficacy of Crizotinib in Patients with Advanced or Metastatic ROS1-Rearranged Lung Cancer (EUCROSS): A European Phase II Clinical Trial. J. Thorac. Oncol..

[14] Shaw A.T., Gandhi L., Gadgeel S., Riely G.J., Cetnar J., West H., Camidge D.R., Socinski M.A., Chiappori A., Mekhail T. (2016). Alectinib in *ALK*-positive, crizotinib-resistant, non-small-cell lung cancer: A single-group, multicentre, phase 2 trial. Lancet Oncol..

[15] Shaw A.T., Kim T.M., Crinò L., Gridelli C., Kiura K., Liu G., Novello S., Bearz A., Gautschi O., Mok T. (2017). Ceritinib versus chemotherapy in patients with ALK-rearranged non-small-cell lung cancer previously given chemotherapy and crizotinib (ASCEND-5): A randomised, controlled, open-label, phase 3 trial. Lancet Oncol..

[16] Stinchcombe T.E. (2021). Lorlatinib in the treatment of anaplastic lymphoma kinase-positive non-small-cell lung cancer. Ann. Oncol..

[17] Brown E.G. (2004). Using MedDRA: Implications for risk management. Drug Saf..

[18] Zhou Z.-X., Yin X.-D., Zhang Y., Shao Q.-H., Mao X.-Y., Hu W.-J., Shen Y.-L., Zhao B., Li Z.-L. (2022). Antifungal Drugs and Drug-Induced Liver Injury: A Real-World Study Leveraging the FDA Adverse Event Reporting System Database. Front. Pharmacol..

[19] Shi A., Nguyen H., Kuo C.B., Beringer P.M. (2024). Drug-induced liver injury associated with elexacaftor/tezacaftor/ivacaftor: A pharmacovigilance analysis of the FDA adverse event reporting system (FAERS). J. Cyst. Fibros..

[20] Caster O., Aoki Y., Gattepaille L.M., Grundmark B. (2020). Disproportionality Analysis for Pharmacovigilance Signal Detection in Small Databases or Subsets: Recommendations for Limiting False-Positive Associations. Drug Saf..

[21] Moyer A.M., Matey E.T., Miller V.M. (2019). Individualized medicine: Sex, hormones, genetics, and adverse drug reactions. Pharmacol. Res. Perspect..

[22] Yu Y., Chen J., Li D., Wang L., Wang W., Liu H. (2016). Systematic Analysis of Adverse Event Reports for Sex Differences in Adverse Drug Events. Sci. Rep..

[23] Chen P., Liu Y., Wen Y., Zhou C. (2022). Non-small cell lung cancer in China. Cancer Commun..

[24] Mangoni A.A., Jackson S.H. (2004). Age-related changes in pharmacokinetics and pharmacodynamics: Basic principles and practical applications. Br. J. Clin. Pharmacol..

[25] Barnes S., Ishii T., Iwasawa S., Kurimoto R., Maeda A., Takiguchi Y., Kaneda M. (2015). Crizotinib-Induced Abnormal Signal Processing in the Retina. PLoS ONE.

[26] Luo Y., Zhang Z., Guo X., Tang X., Li S., Gong G., Gao S., Zhang Y., Lin S. (2023). Comparative safety of anaplastic lymphoma kinase tyrosine kinase inhibitors in advanced anaplastic lymphoma kinase-mutated non-small cell lung cancer: Systematic review and network meta-analysis. Lung Cancer.

[27] Xu Z., Pan Z., Jin Y., Gao Z., Jiang F., Fu H., Chen X., Zhang X., Yan H., Yang X. (2023). Inhibition of PRKAA/AMPK (Ser485/491) phosphorylation by crizotinib induces cardiotoxicity via perturbing autophagosome-lysosome fusion. Autophagy.

[28] Hwang J., Lee Y., Kang K., Eun M.Y. (2023). Acute Ischemic Stroke Caused by Intracranial Atherosclerosis Associated With Lorlatinib-Induced Dyslipidemia. J. Clin. Neurol..

[29] Sato J., Uchida M., Wakabayashi H., Shimizu T. (2022). Evaluation of Lung Toxicity Related to the Treatment With Alectinib Using a Pharmacovigilance Database. Anticancer. Res..

[30] Maideen N.M.P., Balasubramanian R., Muthusamy S. (2022). A Comprehensive Review of the Pharmacologic Perspective on Loop Diuretic Drug Interactions with Therapeutically Used Drugs. Curr. Drug Metab..

[31] Misawa K., Nakamichi S., Iida H., Nagano A., Mikami E., Tozuka T., Matsumoto M., Miyanaga A., Noro R., Kubota K. (2023). Alectinib-Induced Severe Hemolytic Anemia in a Patient with ALK-Positive Non-Small Cell Lung Cancer: A Case Report. OncoTargets Ther..

[32] Li J., Yuan Z., Wang Q., Fan W., Zhang G. (2019). Meta-analysis of overall incidence and risk of ALK inhibitors-induced liver toxicities in advanced non-small-cell lung cancer. Medicine.

[33] Nishio M., Yoshida T., Kumagai T., Hida T., Toyozawa R., Shimokawaji T., Goto K., Nakagawa K., Ohe Y., Seto T. (2021). Brigatinib in Japanese Patients With ALK-Positive NSCLC Previously Treated With Alectinib and Other Tyrosine Kinase Inhibitors: Outcomes of the Phase 2 J-ALTA Trial. J. Thorac. Oncol..

