# Supplementary material for: Safety Profile and Hepatotoxicity of Anaplastic Lymphoma Kinase Tyrosine Kinase Inhibitors: A Disproportionality Analysis Based on FDA Adverse Event Reporting System Database
Source: Toxics. 2025 Mar 14;13(3):210. doi: 10.3390/toxics13030210 (PMC11946249; doi:10.3390/toxics13030210)
Supplement: Supplementary file 1 [file toxics-13-00210-s001.zip › toxics-3509292-supplementary.pdf]

Supplementary information

Table S1. Dictionary of preferred terms for liver injury

preferred terms (PT)
alanine aminotransferase abnormal
alanine aminotransferase increased
ammonia increased
aspartate aminotransferase abnormal
aspartate aminotransferase increased
bile duct damage
biliary cholangitis
bilirubin conjugated increased
bilirubin urine
blood bilirubin abnormal
blood bilirubin increased
blood bilirubin unconjugated increased
cholestasis
cirrhosis
coma hepatic
dili
drug-induced liver injury
hepatic cirrhosis
hepatic damage
hepatic disease
hepatic encephalopathy
hepatic enzyme abnormal
hepatic enzyme increased
hepatic failure
hepatic function abnormal
hepatic injury
hepatic necrosis
hepatic steatosis
hepatic vascular injury
hepatitis
hepatobiliary disease
hepatocellular damage
hepatocellular injury
hepatomegaly
hepatopathy
hepatotoxicity
hyperammonaemia

hyperbilirubinaemia
icterus
jaundice
liver damage
liver fatty infiltration
liver function test abnormal
liver injury
liver necrosis
liver transplant
mixed hepatocellular-cholestatic injury
nonalcoholic fatty liver disease
portal hypertension
steatohepatitis
transaminases abnormal
transaminases increased
urine bilirubin increased

Table S2. Contingency table

	Target AEs	Non-target AEs	Total
Target drug	a	b	a+b
Non-target drug	c	d	c+d
Total	a+c	b+d	N=a+b+c+d

Table S3. ROR, PRR, BCPNN, and EBGM methods, formulas, and thresholds

Method	Formula	Standard Threshold
ROR	$ROR=(a/c)/(b/d)$<br>$SE\ (lnROR)=\sqrt{(1/a+1/b+1/c+1/d)}$<br>$95\%CI=e^{\wedge}\ (ln\ (ROR)\pm 1.96\sqrt{(1/a+1/b+1/c+1/d)})$	a≥3 and 95% CI (lower limit)> 1
PRR	$PRR=(a/(a+b))/(c/(c+d))$<br>$\chi^{\wedge}2=((ad-bc)\ ^{\wedge}\ 2\ (a+b+c+d))/((a+b)\ (a+c)\ (c+d)\ (b+d))$	a ≥ 3 and PRR value ≥ 2
BCPNN	$IC=log_2(p(x,y))/(p(x)p(y))=log_2(a(a+b+c+d))/((a+b)(a+c))$<br>$E(IC)=log_2((a+\gamma 11)(a+b+c+d+\alpha)(a+b+c+d+\beta))/((a+b+c+d+\gamma)(a+b+c+d+\beta 1))$<br>$V(IC)=(1/(ln2)^{\wedge}2)\{[((a+b+c+d)-\alpha+\gamma-\gamma 11)/((a+\gamma 11)(1+a+b+c+d+\gamma))]+[((a+b+c+d)-(a+b)+\alpha-\alpha 1)/((a+b+\alpha 1)(1+a+b+c+d+\alpha))]+[((a+b+c+d)-(a+c)+\beta-\beta 1)/((a+c+\beta 1)(1+a+b+c+d+\beta))]\}$<br>$\gamma=\gamma 11\ ((a+b+c+d+\alpha)\ (a+b+c+d+\beta))/((a+b+\alpha 1)\ (a+c+\beta 1))$<br>$IC\text{-}2SD=E(IC)-2\sqrt{(V(IC))}$	IC025>0
EBGM	$EBGM=(a(a+b+c+d))/((a+c)\ (a+b))$<br>$95\%CI=e^{\wedge}\ (ln\ (EBGM)\pm 1.96\sqrt{(1/a+1/b+1/c+1/d)})$	EBGM05>2

ROR Reporting Odds Ratio, *PRR* Proportional Reporting Ratio, *IC* Information Component, *EBGM* Empirical Bayesian

Geometric Mean.

Table S4. Common adverse events signals for five drugs

PT
dyspnoea
pleural effusion
dysphagia
renal impairment
pneumonitis
blood creatinine increased
respiratory failure
aspartate aminotransferase increased
hepatic function abnormal
pericardial effusion
liver function test increased
lung disorder
haemoptysis
carcinoembryonicantigen increased
brain oedema

Table S5. Basic information for DILI of crizotinib, ceritinib, alectinib, brigatinib, lorlatinib

Characteristics	crizotinib	ceritinib	alectinib	brigatinib	lorlatinib
**Total number**	696	451	528	178	125
**Age (years), mean±SD**	57.11±16.64	53.78±17.83	56.98±14.12	60.74±12.95	53.01±16.30
**Sex**					
Female	355 (51.01)	244 (54.10)	278 (52.65)	105 (58.99)	55 (44.00)
Male	272 (39.08)	181 (40.13)	193 (36.55)	67 (37.64)	62 (49.60)
**Unknown**	69 (9.91)	26 (5.76)	57 (10.80)	6 (3.37)	8 (6.40)
**Reporter country**					
United states	280 (40.23)	62 (13.75)	162 (30.68)	38 (21.35)	22 (17.60)
Japan	106 (15.23)	106 (23.50)	58 (10.98)	63 (35.39)	14 (11.20)
China	25 (3.59)	30 (6.65)	48 (9.09)	29 (16.29)	14 (11.20)
Other	285 (40.95)	253 (56.10)	260 (49.24)	48 (26.97)	75 (60.00)
**Reporter**					

Physician	415 (59.63)	228 (50.55)	305 (57.77)	96 (53.93)	53 (42.40)
Consumer	110 (15.80)	94 (20.84)	110 (20.83)	40 (22.47)	39 (31.20)
Pharmacist	51 (7.33)	13 (2.88)	44 (8.33)	19 (10.67)	9 (7.20)
Other health professionals	116 (16.67)	87 (19.29)	68 (12.88)	23 (12.92)	24 (19.20)
Unknown	4 (0.57)	29 (6.43)	1 (0.19)	0 (0)	0 (0)
**Outcome**					
Death	118 (16.95)	80 (14.74)	25 (4.73)	13 (7.30)	20 (16.00)
Hospitalization-Initial or prolonged	150 (21.55)	121 (26.83)	95 (17.99)	59 (33.15)	33 (26.40)
Life threatening	24 (3.45)	15 (3.33)	24 (4.55)	1 (0.56)	0 (0)
Disability	5 (0.72)	4 (0.89)	12 (2.27)	0 (0)	1 (0.80)
Othesr serious outcome	247 (35.49)	198 (43.90)	288 (54.55)	90 (50.56)	58 (46.40)
Unknown	152 (21.84)	33 (7.32)	84 (15.91)	15 (8.43)	13 (10.40)
**Adverse event occurrence time-medication date (days)**					
0-7	0 (0)	1 (0.22)	22 (4.17)	3 (1.69)	4 (3.20)
7-28	6 (0.86)	7 (1.55)	9 (1.70)	22 (12.36)	5 (4.00)
28-60	12 (1.72)	5 (1.11)	15 (2.84)	3 (1.69)	2 (1.60)
≥60	7 (1.01)	16 (3.55)	12 (2.27)	8 (4.49)	12 (9.60)
Unknown	671 (96.41)	422 (93.57)	470 (89.02)	142 (79.78)	102 (81.60)

Table S6. Signal analysis of DILI in ALK-TKIs

PT	N	ROR (95% CI)	PRR(χ2)	IC(IC025)	EBGM(EBGMO5)
alanine aminotransferase increased	216	5.65(4.95-6.47)	5.63(819.84)	2.49(0.82)	5.61(5.02)
aspartate aminotransferase increased	206	6.52(5.68-7.48)	6.49(953.29)	2.69(1.03)	6.47(5.76)
hepatic enzyme increased	172	3.72(3.2-4.32)	3.71(340.12)	1.89(0.22)	3.7(3.27)
hepatic function abnormal	164	6.63(5.68-7.73)	6.61(776.87)	2.72(1.05)	6.58(5.78)
transaminases increased	109	7.25(6-8.75)	7.23(581.98)	2.85(1.18)	7.19(6.14)
liver function test abnormal	88	4.95(4.02-6.11)	4.94(275.91)	2.3(0.64)	4.93(4.14)

liver function test increased	81	5.12(4.12-6.37)	5.11(266.95)	2.35(0.68)	5.1(4.24)
hepatotoxicity	75	5.09(4.06-6.39)	5.08(245.18)	2.34(0.68)	5.07(4.19)
blood bilirubin increased	65	3.85(3.02-4.92)	3.85(136.75)	1.94(0.28)	3.84(3.13)
hepatitis	63	4.11(3.21-5.27)	4.11(147.67)	2.03(0.37)	4.1(3.33)
hepatocellular injury	51	4.55(3.46-5.99)	4.55(140.61)	2.18(0.51)	4.53(3.6)
hepatitis fulminant	16	8.91(5.45-14.57)	8.91(111.51)	3.15(1.48)	8.85(5.86)
hepatitis acute	15	3.96(2.39-6.58)	3.96(33.12)	1.98(0.32)	3.95(2.59)
hepatic lesion	12	3.41(1.94-6.01)	3.41(20.39)	1.77(0.1)	3.4(2.12)
aspartate aminotransferase abnormal	12	12.77(7.23-22.55)	12.76(128.8)	3.66(1.99)	12.65(7.86)
hepatic cyst	11	3.97(2.2-7.18)	3.97(24.39)	1.99(0.32)	3.96(2.41)
alanine aminotransferase abnormal	10	6.2(3.33-11.54)	6.2(43.4)	2.63(0.96)	6.17(3.67)
blood bilirubin unconjugated increased	4	10.38(3.88-27.77)	10.38(33.62)	3.36(1.69)	10.3(4.52)
hepatobiliary disease	3	6.16(1.98-19.17)	6.16(12.91)	2.62(0.95)	6.14(2.38)
blood bilirubin decreased	3	6.22(2-19.35)	6.22(13.09)	2.63(0.96)	6.2(2.4)

[a] indicates a PT not yet mentioned in the drug instruction. *PT* Preferred Term in MedDRA, *ROR* Reporting Odds Ratio, *PRR* Proportional Reporting Ratio, *IC* Information Component, *EBGM* Empirical Bayesian Geometric Mean

Table S7. Label w~~W~~arnings and h~~H~~epatic s~~S~~afety g~~G~~uidelines for ALK-TKIs

Drug	Label Warnings	Dose Adjustments for Hepatic Impairment	Dose Adjustments for ALT/AST Elevation	Liver Monitoring Guidelines
crizotinib	-Severe hepatotoxicity<br>-Monitor liver function during therapy	-Mild/Moderate (Child-Pugh A/B): No adjustment.<br>-Severe (Child-Pugh C): Avoid use.	-ALT/AST >5× ULN: Withhold therapy; resume at reduced dose (200 mg BID) if recovered to ≤3× ULN.<br>-Recurrent elevation: Discontinue.	-Baseline ALT/AST/bilirubin.<br>Monitor every 2 weeks for 2 months, then monthly.
ceritinib	-Severe hepatotoxicity<br>-Baseline and periodic liver function monitoring required	-Mild: No adjustment.<br>-Moderate/Severe: Reduce dose to 300 mg QD or avoid.	-ALT/AST >5× ULN: Withhold; resume at 450 mg QD if recovered to ≤3× ULN.<br>-Recurrence: Discontinue.	-Baseline and monthly ALT/AST/bilirubin.<br>Increase to weekly if abnormalities occur.
alectinib	-Risk of ALT/AST elevation<br>-Use caution in severe hepatic impairment	-Mild/Moderate: No adjustment.<br>-Severe: Limited data; use with caution.	-ALT/AST >5× ULN: Withhold; resume at 450 mg BID if recovered to ≤3× ULN.<br>-Persistent elevation: Discontinue.	-Baseline and every 2 weeks for 3 months, then periodically.
brigatinib	-Low hepatotoxicity risk<br>-monitor liver enzymes	-Mild/Moderate: No adjustment.<br>-Severe: Avoid use.	-ALT/AST >5× ULN: Withhold; resume at 90 mg QD if recovered to ≤3× ULN.<br>-Recurrence: Discontinue.	-Baseline and periodic monitoring (suggested monthly).
lorlatinib	-ALT/AST elevation<br>-Rare severe hepatotoxicity	-Mild/Moderate: No adjustment.<br>-Severe: Insufficient data; use caution.	-ALT/AST >5× ULN: Withhold; resume at 75 mg QD if recovered to ≤3× ULN.<br>-Recurrence: Discontinue.	-Baseline and every 2 weeks for 3 months, then monthly.

Table S8. Global approval timeline of ALK-TKIs

Drug	USA (FDA)	EU (EMA)	Japan (PMDA)	China (NMPA)	Other Major Markets
crizotinib	2011	2012	2012	2013	Canada:2012; Australia: 2013
ceritinib	2014	2015	2014	2018	South Korea: 2015; Switzerland: 2015
alectinib	2015	2017	2014	2018	Canada: 2016; Brazil: 2017
brigatinib	2017	2018	2019	2020	Australia: 2018; India: 2019
lorlatinib	2018	2019	2020	2022	Canada: 2019; South Korea: 2020

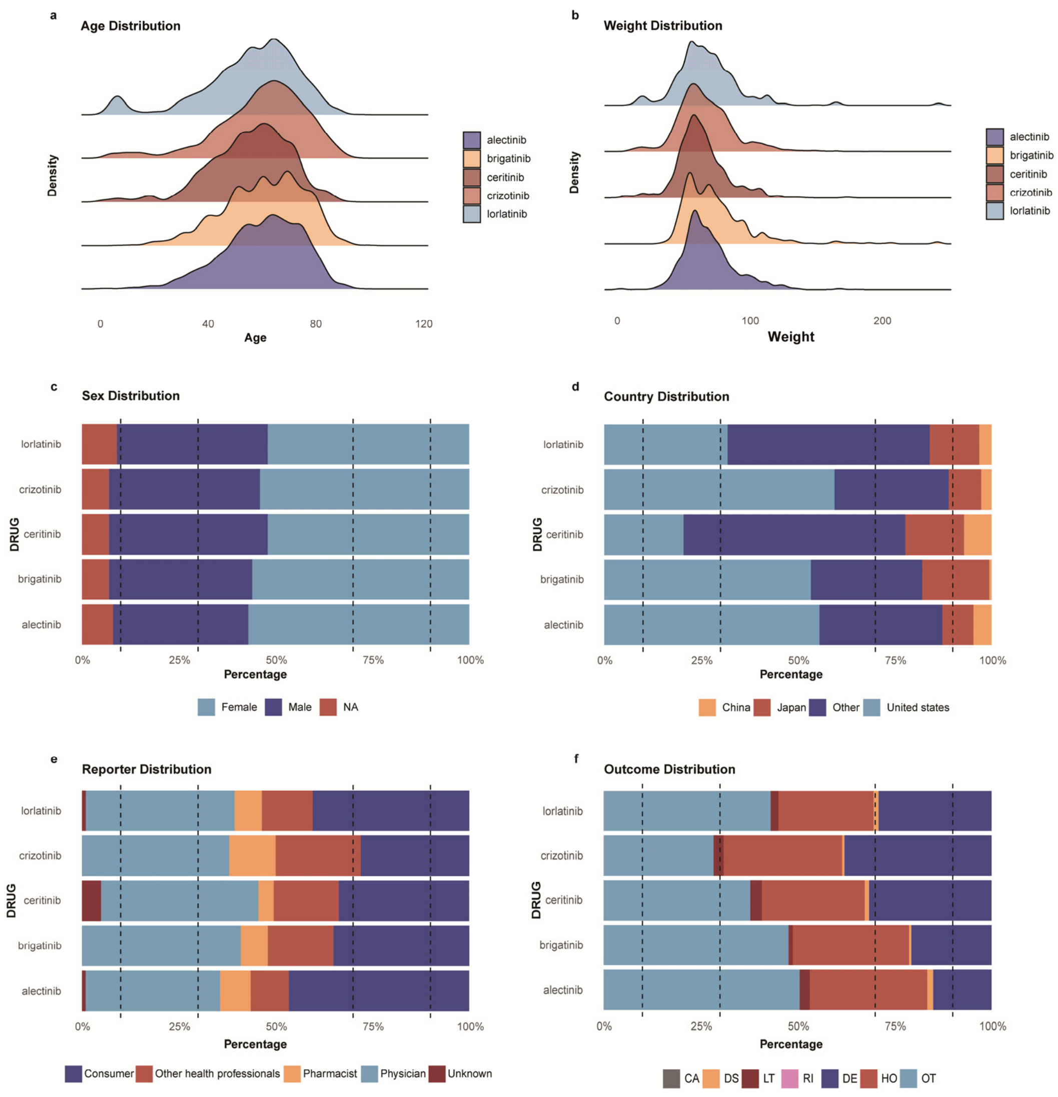

Figure S1. Distribution of age(a), weight(b), sex(c), country of reporter(d), the type of reporter(e), and outcome(f) in reports for five drugs. *CA* congenital anomaly, *DS* disability, *LT* life-threatening, *RI* required intervention to prevent permanent impairment or damage, *DE* death, *HO* hospitalization initial or prolonged, *OT* other serious (important medical event)

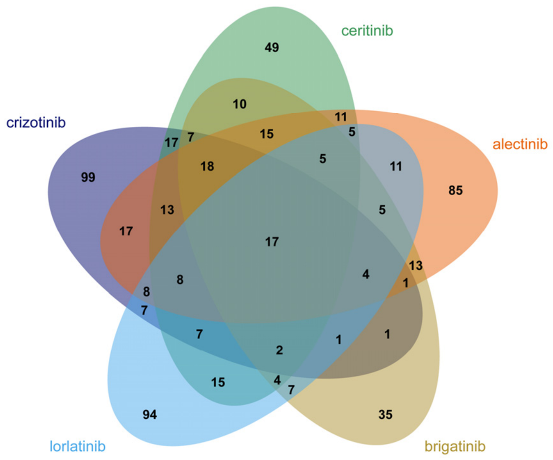

Figure S2. Wayne plots of adverse event signals for the five drugs
